# Hollow-SiO_2_@Cu_*x*_Zn_*y*_Mg_*z*_Al-LDHs as catalyst precursors for CO_2_ hydrogenation to methanol†

**DOI:** 10.1039/d4sc07292h

**Published:** 2024-12-17

**Authors:** Tomasz Kondratowicz, Marta Gajewska, Jiangtong Li, Molly Meng-Jung Li, Zoë R. Turner, Chunping Chen, Dermot O'Hare

**Affiliations:** a Chemistry Research Laboratory, Department of Chemistry, University of Oxford 12 Mansfield Road Oxford OX1 3TA UK chunping.chen@chem.ox.ac.uk dermot.ohare@chem.ox.ac.uk +44 (0)1865 272686; b Academic Centre for Materials and Nanotechnology, AGH University of Krakow Mickiewicza 30 30-059 Krakow Poland; c Department of Applied Physics, The Hong Kong Polytechnic University P. R. China

## Abstract

We report a new synthetic strategy for preparing well-organised, spherical and mesoporous, mixed-metal, hollow-core@layered double hydroxides. Hollow-SiO_2_@Cu_*x*_Zn_*y*_Mg_*z*_Al-LDHs (*x* + *y* + *z* = 2.32 ± 0.06) were prepared by exploiting a unique “memory effect” feature of LDH materials. The reconstruction with simultaneous incorporation of Cu^2+^ and Zn^2+^ into the LDH shell was achieved by exposing hollow-SiO_2_@Mg_2_Al-LDO to an aqueous solution containing Cu^2+^ and Zn^2+^ cations. The effect of a single reconstruction step with various concentrations of Cu^2+^ and Zn^2+^ solutions (20–80 mM), as well as the implementation of five successive cycles of calcination–reconstruction on the chemical composition, morphology, texture and structure of the resulting materials are described. Hollow-SiO_2_@Cu_*x*_Zn_*y*_Mg_*z*_Al-LDHs are precursors to active catalysts for CO_2_ hydrogenation to methanol. The most active catalyst exhibits a space-time yield for methanol of 1.68 g_MeOH_ g_Cu_^−1^ h^−1^ at 270 °C (3 : 1 CO_2_ : H_2_, 30 bar) which represents a 1.7-fold increase in space-time yield compared to commercial Cu/ZnO/Al_2_O_3_ catalyst under the same conditions.

## Abstract 2

We report a new synthetic strategy for preparing well-organised, spherical and mesoporous, mixed-metal, hollow-core@layered double hydroxides.

## Graphical abstract

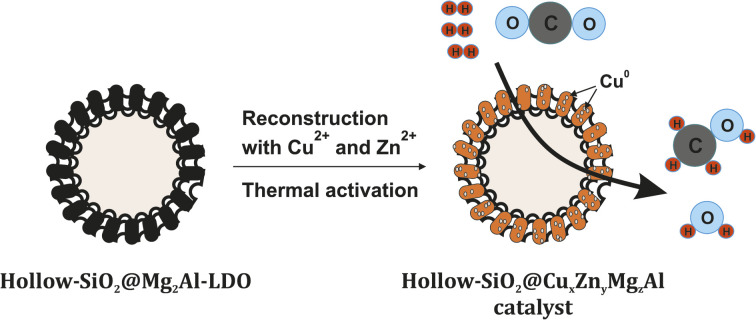

## Introduction

Over several decades, rapid development of civilisation and industrial expansion have sharply increased carbon dioxide (CO_2_) emissions, reaching approximately 35 billion tons in 2021.^1^ This has led to serious environmental issues including global warming, acid rain, ocean acidification and rising sea levels. The primary challenge now is to mitigate anthropogenic CO_2_ emissions by (i) improving energy efficiencies to reduce CO_2_ production, and (ii) implementing carbon capture and storage (CCS) and/or carbon capture and utilisation (CCU) technologies to manage the emissions effectively.^2,3^ CCS technology typically involves capturing CO_2_ from gas streams (*e.g.*, flue gas) followed by storing it underground. However, this requires high investment and poses risks such as potential leakage of CO_2_.^4^ Unlike CCS, CCU upgrades CO_2_ waste into valuable products, such as polymers, chemicals and fuels.^3–5^ In particular, methanol has been proposed as a promising product from CO_2_ hydrogenation. Methanol has the potential to play a dual role in both the energy and chemicals sectors as highlighted by “methanol economy” concept.^6^ It can be used directly as fuel or blended with conventional fuels. It is also a key feedstock for the production of chemicals such as formaldehyde, dimethyl ether and acetic acid.^7^ As one of the ten most important industrial chemicals, global methanol production exceeded 110 million tons in 2018, with expectation to double by 2030.^8^ Since the 1960s, methanol has been produced on an industrial scale from CO-rich syn-gas (CO/CO_2_/H_2_) generated from fossil fuels, using a Cu/ZnO/Al_2_O_3_ catalyst at 3–5 MPa pressure and 200–300 °C.^9^ This well-established process can be readily used for the CO_2_ hydrogenation to produce green methanol, using captured CO_2_ and green hydrogen. Such approach has already been demonstrated at pilot-scale plants in Europe and Asia.^2,10,11^

It is widely accepted that metallic copper (Cu^0^) produces the active sites for CO_2_ hydrogenation to methanol, with a well-established relationship between the addressable specific Cu^0^ surface area and catalyst performance.^12,13^ The Cu/ZnO/Al_2_O_3_ combination remains the most widely used catalyst system for methanol production *via* CO_2_ hydrogenation. ZnO acts as Cu promoter which can enhance and stabilise the Cu dispersion and adsorption of CO_2_, while Al_2_O_3_ can enhance thermal and chemical stability. In addition to these core components, various promoters such as Cr, Ga, Zr, Mg, Sr, Ba, and others have been extensively studied to improve catalytic performance by enhancing Cu dispersion, reducing Cu^0^ particle size, increasing Cu stability and reducibility.^14–18^ Among these, Mg has garnered particular attention due to its ability to modify the basicity for CO_2_ adsorption and stabilise the Cu/ZnO/Al_2_O_3_ interface. Recently, layered double hydroxides (LDHs) have been shown to offer great potential as precursors in making robust Cu/Zn_*x*_AlO_*y*_ catalysts. Excellent metal dispersions are achieved by the incorporation of Cu, Zn, and Al into the LDH metal hydroxide layers.^19–21^ LDHs are a large family of anionic layer materials, the most frequently observed formulation can be written as [M^II^_1−*x*_M^III^_*x*_(OH)_2_]^*x*+^[(A^*n*−^)_*x*/*n*_]·*m*H_2_O, consisting of positively-charged mixed metal hydroxide layers ([M^II^_1−*x*_M^III^_*x*_(OH)_2_]^*x*+^) intercalated with hydrated charge compensating anions ([(A^*n*−^)_*x*/*n*_·*m*H_2_O]). LDHs offer advantages such as compositional flexibility and atomic-scale dispersion of metal ions within the layers.^22^ Upon calcination between 300–600 °C, LDHs transform into homogeneous mixed metal oxides, also known as layered double oxides (LDOs),^23^ which exhibit an unique “memory effect” that may allow reconstruction of the original layered structure in the presence of water, either in the gas or liquid phase.^24,25^ This behavior has allowed incorporation of new metal cations into the LDH matrix, simply *via* exposing the LDO to aqueous cations solutions.^26–28^ Despite the well-established, cost-effective methods for LDH synthesis, conventional techniques often result in aggregated powders with poor morphology and irregular particle size (so-called stone-like morphology), limiting their porosity and practical applications.^29^ One approach to mitigate these limitations is the decoration of LDH platelets on well-defined core inorganic or organic supports, *e.g.* SiO_2_,^30^ Fe_*x*_O_*y*_,^31^ TiO_2_,^32^ Cu_2_O,^33^ zeolites,^34^ MOF^35^ and carbon^36^ to create core–shell hybrids with shapes including spheres,^30^ cubes,^33^ wires^37^ and rods.^38^ Notably, core–shell structures typically contain vertically oriented LDH platelets forming a three-dimensional (3D) honeycomb-like LDH shell that exhibits enhanced textural properties and active site accessibility compared to unsupported LDH materials.^39,40^ Additionally, such hybrids can serve as templates for hollow structures,^40–42^ which have demonstrated enhanced properties over the parent core–shell materials such as higher porosity and concentration of basic sites.^42^

Previously, we have reported the synthesis of silica@Cu_*x*_ZnAl-LDH (*x* = 0.8–4.0) core–shell hybrids, using commercially available ES757 silica, mesoporous MCM-48, and SBA-16 as a core, with various Cu_*x*_ZnAl-LDHs used as a shell.^20^ Upon thermal activation, these core–shell materials exhibited significantly improved catalytic performance for CO_2_ hydrogenation to methanol compared to their equivalent unsupported Cu_*x*_ZnAl LDH precursors. Hollowed core@shell materials offer the advantage of greater catalytic activity per mass of catalyst as a result of reduction in inactive core, as well as potential for improved gas diffusion through the catalyst. Herein, we report a new advance by developing a novel strategy for the synthesis of hollow-SiO_2_@Cu_*x*_Zn_*y*_Mg_*z*_Al-LDH catalyst precursors for CO_2_ hydrogenation to methanol.

## Results and discussion

### Synthetic strategy to obtain hollow-SiO_2_@Cu_*x*_Zn_*y*_Mg_*z*_Al-LDHs

**Fig. 1:**
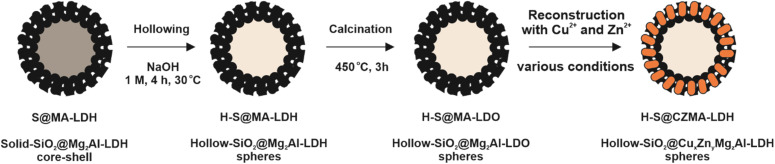
Synthetic scheme used to obtain mixed-metal (Cu, Zn) hollow-SiO_2_@Cu_*x*_Zn_*y*_Mg_*z*_Al-LDH spheres.

The concept for developing mixed-metal hollow-SiO_2_@Cu_*x*_Zn_*y*_Mg_*z*_Al-LDHs is schematically shown in Fig. 1. In the first step, solid SiO_2_@Mg_2_Al-LDH core–shell particles (S@MA-LDH) were synthesised by the pH controlled coprecipitation of Mg_2_Al–CO_3_ LDH in the presence of non-porous spherical SiO_2_ particles. The silica core from SiO_2_@Mg_2_Al-LDH was then selectively etched under static conditions by treatment with a NaOH solution to yield hollow-SiO_2_@Mg_2_Al-LDH spheres (H-S@MA-LDH), followed by calcination to produce hollow-SiO_2_@Mg_2_Al-LDO spheres (H-S@MA-LDO), according to our previous protocol.^42^ Afterwards, the hollow-SiO_2_@Mg_2_Al-LDO spheres were added to aqueous Cu and Zn nitrate solutions, facilitating ion exchange between the transition metals and Mg^2+^ cations during reconstruction of the Mg_2_Al-LDO into Cu_*x*_Zn_*y*_Mg_*z*_Al-LDH through the memory effect, to obtain hollow-SiO_2_@Cu_*x*_Zn_*y*_Mg_*z*_Al-LDH (H-S@CZMA-LDH_X, where X depends on applied reconstruction conditions). The impact of a single reconstruction step using various concentrations of Cu and Zn solutions, as well as the implementation of repeated calcination and reconstruction cycles on the chemical composition, morphology, structural and textural properties of the hollow sphere materials has been studied.

### Synthesis of hollow-SiO_2_@Mg_2_Al-LDH spheres

**Fig. 2:**
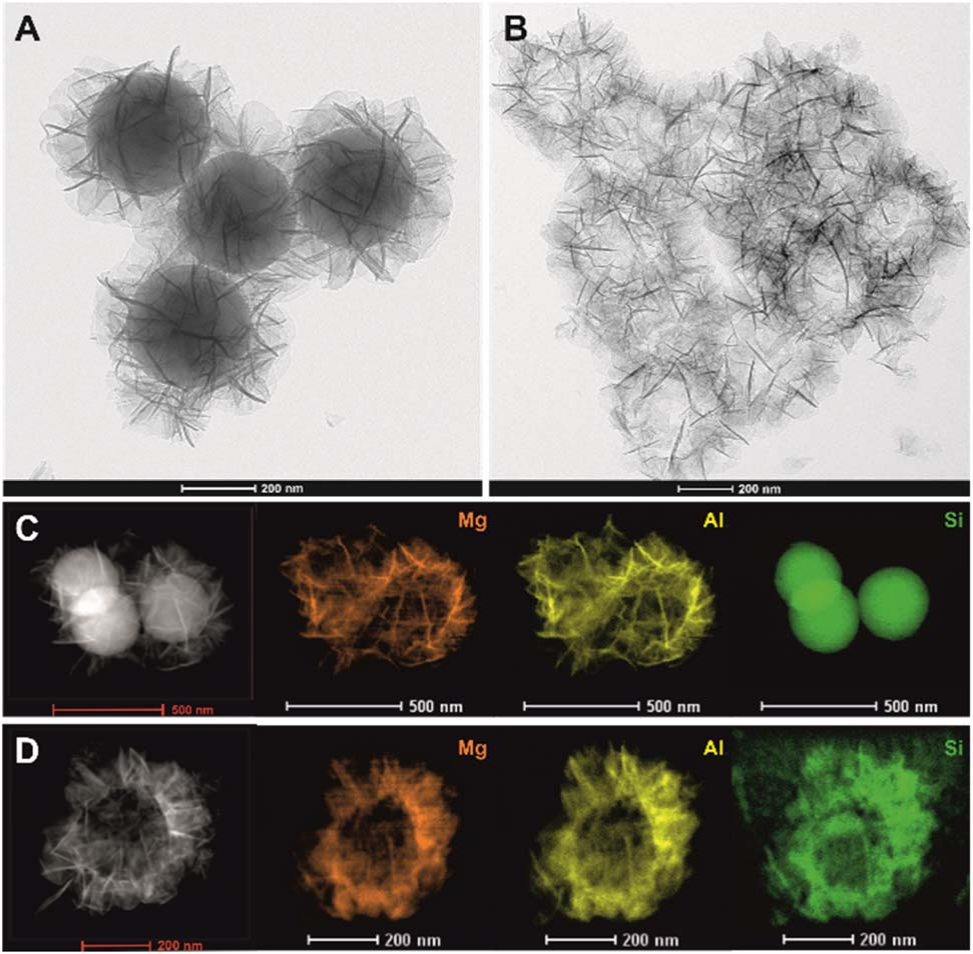
TEM images of S@MA-LDH solid core–shell (A), H-S@MA-LDH hollow spheres (B) and Mg, Al, Si elemental EDX mapping of S@MA-LDH (C) and H-S@MA-LDH (D).

Fig. 2 shows the transmission electron microscope (TEM) images of pristine solid S@MA-LDH core–shell and the H-S@MA-LDH hollow spheres obtained after the leaching of the SiO_2_ core. It can be clearly observed that sample before etching (Fig. 2A) exhibits typical core–shell structure, with a dark solid core sphere surrounded by a bright shell composed of vertically grown hierarchical nanosheets of LDH. The average diameter of the core–shell particles is 562 ± 25 nm, of which the SiO_2_ core diameter is 340 ± 9 nm and the LDH shell thickness is 112 ± 22 nm, consistent with S@MA-LDH prepared previously.^37^ The EDX elemental maps in Fig. 2C clearly demonstrate that Si is mainly distributed in the core while Mg and Al are uniformly dispersed across the shell, confirming the formation of core–shell structure with SiO_2_ as core and Mg_2_Al-LDH as shell. After 4 h of immersion in an alkali solution, the SiO_2_ core was removed as evidenced in Fig. 2B, resulting in hollow shell structure with a clearly visible void inside the sphere while retaining the original particle size and spherical shape of the parent S@MA-LDH. The disappearance of contrast between the core and the shell confirms the high efficiency of the core removal. The elemental mapping analysis (Fig. 2D) further support this, as Si is absent in the core region but appears in the shell, likely due to partial deposition of Si species after leaching process.

The elemental compositions were further quantitatively analysed using elemental microanalysis (CHN), energy dispersive X-ray spectroscopy (EDX), inductively coupled plasma optical emission spectroscopy (ICP-OES) and thermogravimetric analysis (TGA). As shown in Table S1,† both EDX and ICP data reveal that Mg/Al molar ratio remained similar before and after the leaching process. However, the molar ratio of Si/Al significantly drops from 3.77 to 0.68, indicating that most SiO_2_ has been removed. This is consistent with TGA analysis showing that the SiO_2_ content decreases from 48.00 wt% to 17.07 wt%. The presence of SiO_2_ in the hollow spheres, even at longer leaching times (up to 48 h) or at much harsh leaching conditions (2 M, 50 °C, 20 h),^42^ is likely related to the formation of Si–O–Al (Mg) bonds.^43^ Using the EDX, TGA and CHN data, we can estimate the chemical formula of S@MA-LDH and H-S@MA-LDH as [SiO_2_]_0.81_@[Mg_2.18_Al_1.00_(OH)_6.36_(CO_3_)_0.13_(OH)_0.74_(H_2_O)_3.64_]_0.19_ and [SiO_2_]_0.44_@[Mg_2.16_Al_1.00_(OH)_6.32_(CO_3_)_0.25_(OH)_0.50_(H_2_O)_0.58_]_0.56_, respectively.

The powder X-ray diffraction (XRD) patterns are shown in Fig. S1.† The virgin core–shell sample presents a combination of the characteristic features of both silica (amorphous structure, broad feature at 2*θ* = 22°) and crystalline LDH (Bragg peaks at 2*θ* = 11.5°, 23.2°, 34.8°, 39.3°, 46.7°, 60.8°, and 62.1°), which could be assigned to the (003), (006), (012), (015), (018), (110), and (113) reflections, typical of the double-layered structure in the trigonal *R*3̄*m* space group.^44^ After 4 h of leaching, the broad feature at 2*θ* = 22° disappears, while the positions of the LDH Bragg reflections remain unchanged. However, the relative intensities slightly increase in H-S@MA-LDH, due to its higher proportion of crystalline LDH phase. The unit cell parameters *a*, *c*, LDH basal spacing *d*(003), and the crystallite domain size in the layer stacking direction, *D*_(003)_ and in the *ab*-plane *D*_(110)_ of H-S@MA-LDH are similar to that of S@MA-LDH (Table S2†), which confirms that applied core leaching process presents minimal impact on the structural properties of the LDH phase.

The N_2_ adsorption isotherms of S@MA-LDH and H-S@MA-LDH are shown in Fig. S2,† and the determined textural parameters are listed in Table S3.† The shape of both isotherms is similar to IVa type, according to the IUPAC classification, with H3 hysteresis loops characteristic for mesoporous materials with slit-shape pores.^45^ Importantly, leaching of the predominant amount of the SiO_2_ template from S@MA-LDH leads to an increase in the porosity for the H-S@MA-LDH spheres, the mesopore (*V*_meso_) and total pore (*V*_total_) volume increase from 0.38 to 0.66 cm^3^ g^−1^ and 0.50 to 0.89 cm^3^ g^−1^, respectively. Furthermore, the N_2_ BET surface area (*S*_BET_) doubles from 108 to 224 m^2^ g^−1^.

### Synthesis of hollow-SiO_2_@Cu_*x*_Zn_*y*_Mg_*z*_Al-LDHs through use of the “memory effect”

#### Single immersion

**Fig. 3:**
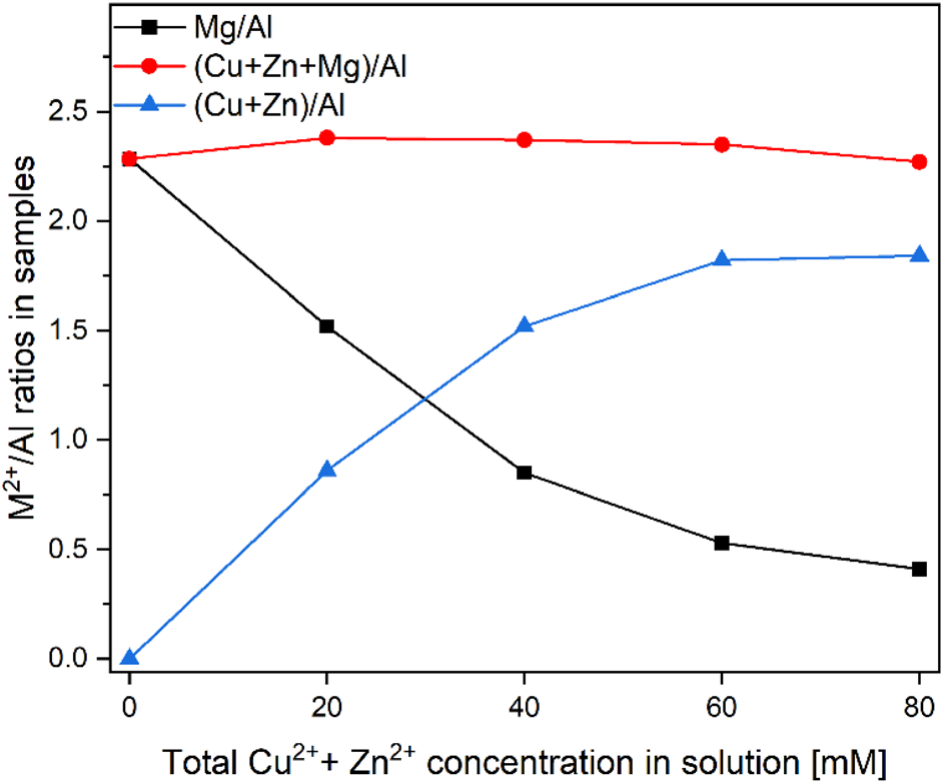
Ratio of Mg/Al, (Cu + Zn + Mg)/Al and (Cu + Zn)/Al in H-S@CZMA-LDHs as a function of the initial aqueous [Cu^2+^] + [Zn^2+^].

It is well-known that LDH materials possess significantly lower cation exchange ability compared to their associated LDO.^46^ Therefore, the H-S@MA-LDH spheres were first calcined (properties of calcined H-S@MA-LDO are summarised in Fig. S3†), and then the M^2+^ : M^3+^ brucite layer composition manipulated through use of the memory effect. The reconstruction and simultaneous incorporation of Cu^2+^ and Zn^2+^ into the brucite layers of the LDH are achieved by treating H-S@MA-LDO with aqueous solutions containing Cu^2+^ and Zn^2+^ cations at total concentrations of 20, 40, 60 and 80 mM (molar ratio of Cu to Zn = 1.30) at room temperature, which promotes a slow rehydration/reconstruction process. The reconstructed samples were designated as H-S@CZMA-LDH_*x* mM, where *x* is the concentration (mM) of the solution used. The elemental composition of the samples before and after a single modification were analysed using the ICP-OES. The results, expressed as Mg/Al, (Cu + Zn + Mg)/Al, and (Cu + Zn)/Al molar ratios, are presented in Fig. 3. A clear trend shows that as the concentration of metal (Cu^2+^ + Zn^2+^) ions increases, the (Cu + Zn)/Al molar ratio in the reconstructed samples gradually increases, reaching values of 0.86, 1.52, 1.82 and 1.84, for 20, 40, 60 and 80 mM, respectively. Conversely, the Mg/Al ratio decreases from 2.28 to 1.52, 0.85, 0.53 and 0.41, respectively. These changes strongly suggest a more extensive exchange of divalent metal ions with higher concentration solutions. Notably, the (Cu + Zn)/Al ratio appears to reach a plateau after modification with 60 mM, indicating that further increasing the concentration of metal solution will not result in additional ion exchange as a maximum has been reached. Interestingly, the (Cu + Zn + Mg)/Al ratio, representing the total M^2+^/Al^3+^ ratio, remains relatively constant (between 2.27 and 2.38), regardless of the metal concentration solutions. Moreover, these values are remarkably close to the initial Mg/Al ratio prior to the reconstruction process (Mg/Al = 2.28), suggesting that the exchange ratio between Mg^2+^ and (Cu^2+^ + Zn^2+^) is nearly equal to 1 : 1.

**Fig. 4:**
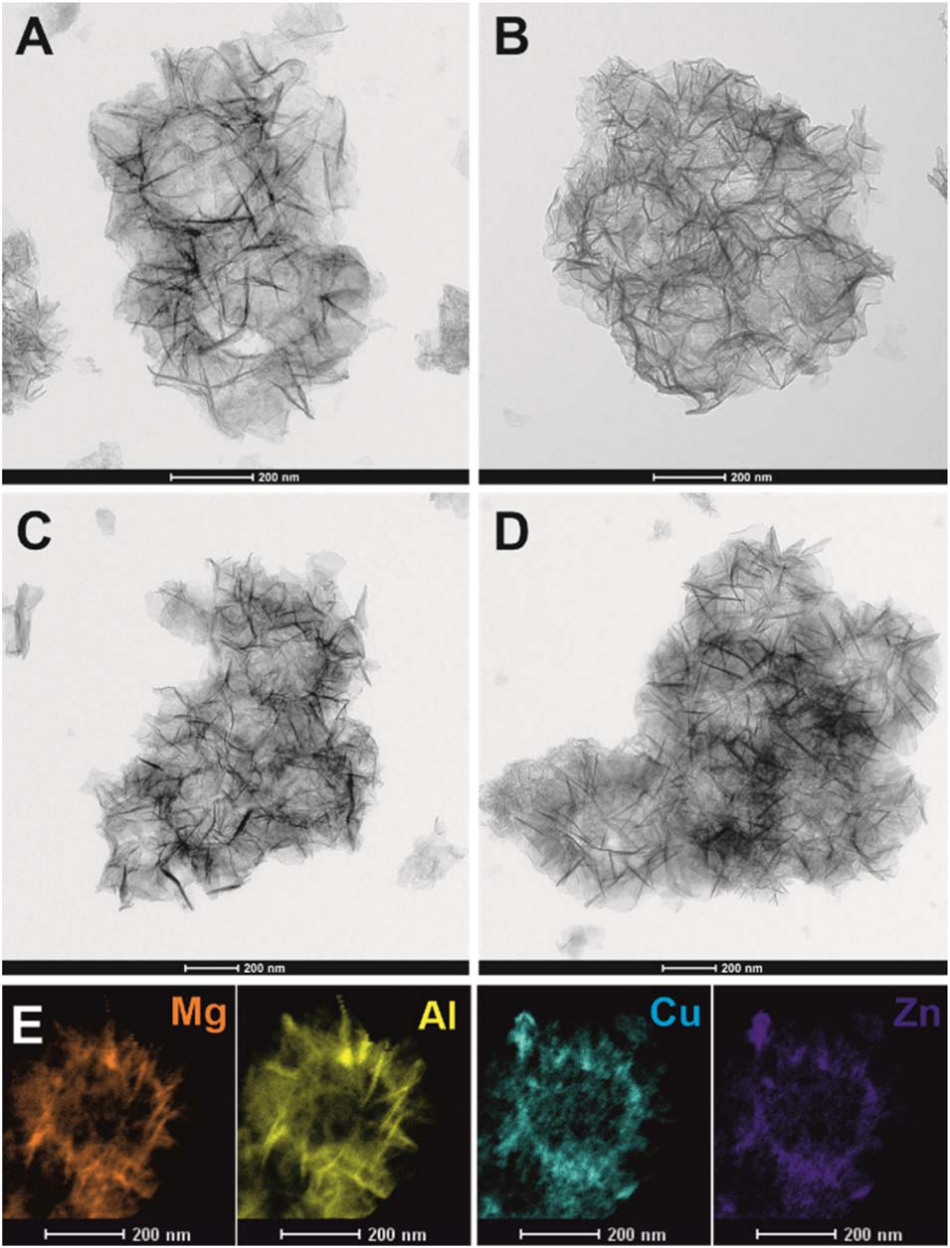
TEM images of H-S@CZMA-LDHs obtained after immersing H-S@MA-LDO in solutions containing Cu^2+^ + Zn^2+^ at a concentration of 20 mM (A), 40 mM (B), 60 mM (C) and 80 mM (D) and Mg, Al, Cu, Zn elemental EDX mapping of H-S@CZMA-LDH_20 mM (E).

Importantly, after LDH reconstruction and simultaneous incorporation of Cu^2+^ and Zn^2+^ into the Mg_*z*_Al layers, the particles still maintain their spherical morphology with a visible hollow core and hierarchical organised platelets on the thin SiO_2_ shell (Fig. 4A–D). A slight morphological degradation occurred when thicker and aggregated particles formed at high metal concentration (80 mM) (Fig. 4D). EDX elemental mapping has been carried out for H-S@CZMA-LDH_20 mM (Fig. 4E); this sample represents an optimised balance between structural integrity with desired spherical morphology and elemental incorporation. A uniform elemental distribution (Mg, Al, Cu, Zn) is observed in the platelets across the spheres, confirming the incorporation of Cu^2+^ and Zn^2+^ ions into the LDH shell layer.

The XRD patterns (Fig. S4†) show that after reconstruction of H-S@MA-LDO in 20 mM solution, characteristic LDH Bragg diffraction features appear. However, when the concentration of the reconstruction solution increased beyond 40 mM, a less crystalline LDH phase becomes apparent, accompanied by a series of intense reflections corresponding to layered metal hydroxy salts (LHS), specifically Cu_2_(OH)_3_NO_3_ (rouaite) with the monoclinic *P*2_1_ space group.^47,48^ The formation of the LHS phase during reconstruction process is consistent with the findings of Wu *et al.*,^46^ who studied the reconstruction of the bulky Mg_3_Al-LDO (Mg/Al = 3) using Zn-nitrate solutions of different concentrations (12–108 mM). The presence of the LHS phase was also detected in samples when the concentration of reconstruction solution reaches 24 mM or higher. Moreover, the intensity of reflections attributed to the LHS phase progressively increases with increasing the concentration of used solutions. A detailed phase analysis using LeBail refinement of the XRD data was performed using TOPAS software (Fig. S5, S6 and Table S4†). Good agreement was obtained using a multi-phase model containing Cu_*x*_Zn_*y*_Mg_*z*_Al-LDH, Cu_2_(OH)_3_NO_3_ and silica. In general, a gradual increase in the content of the LHS phase is observed with the increase in the concentration of CuZn-containing solutions. The content of both LHS and LDH phases in the discussed H-S@CZMAl-LDHs are 14.7, 23.9, 38.6 at% and 73.3, 62.5, 50.1 at%, respectively (Fig. S6†). Moreover, the silica content is around 11.3–13.6 at% which is lower than what we found from EDX result probably due to the existing of amorphous SiO_2_ phase.

The IR spectra of H-S@CZMA-LDHs as shown in Fig. S7† exhibit the characteristic bands of LDH: O–H stretching at 3440 cm^−1^ from the hydroxide group and bending vibration from water molecules (1637 cm^−1^), indicating successful rehydration of LDO through the memory effect. It is worth pointing out that the pure LDH phase is reconstructed with the incorporation of CO_3_^2−^ anions in the interlayer space at low CuZn concentration (20 mM), which is confirmed by the strong absorption band at 1360 cm^−1^ (associated with *ν*_3_ mode of CO_3_^2−^).^49^ However, at higher concentrations of Cu and Zn metal ion solutions (40–80 mM), new bands are observed at 1336 and 1417 cm^−1^, which correspond to the *ν*_3_ mode of NO_3_^−^. Such signals can be assigned to Cu_2_(OH)_3_NO_3_ phase^50,51^ as well as the anion exchanged nitrate in LDH phase. At the same time, the CO_3_^2−^ signal at 1360 cm^−1^ disappears or overlaps with the neighbouring bands, which does not exclude the presence of CO_3_^2−^ ions in the samples.

After the reconstruction and incorporation with Cu^2+^ and Zn^2+^ cations, the adsorption–desorption isotherms (Fig. S8†) of the H-S@CZMA-LDHs remained type IVa with an H3 hysteresis loop according to IUPAC classification. However, the total pore volume and BET surface area (Table S3†) vary. The N_2_*S*_BET_ values for samples immersed in 20 mM and 40 mM solutions are comparable to those of the parent H-S@MA-LDH (225 and 237 *vs.* 224 m^2^ g^−1^) while *V*_total_ slightly decreases (0.81 and 0.78 *vs.* 0.89 cm^3^ g^−1^). However, the samples modified in more concentrated solutions (60 and 80 mM) exhibit a significant reduction in *S*_BET_ and *V*_total_ (156 and 113 m^2^ g^−1^, 0.55 and 0.46 cm^3^ g^−1^, respectively). A similar trend is observed for the mesopore volume which gradually decreases from 0.67 to 0.37 cm^3^ g^−1^ with increasing metal concentration. This porosity reduction is likely due to the increasing presence of the highly crystalline Cu_2_(OH)_3_NO_3_ phase (Fig. S4–S6†). In addition, gradually changing morphology of the samples (Fig. 4) may contribute to partial pore blockage.

#### Calcination–reconstruction cycle

As demonstrated above, a single immersion of H-S@MA-LDO spheres introduces significant amounts of Cu^2+^ and Zn^2+^ ions into LDH layers. However, this is accompanied by a gradual degradation of a hollow shell structure and the formation of undesirable phases at high concentration. To address this, we investigated the effect of repeated calcination–reconstruction cycles (five such repetitions were performed, and the samples were designated as H-S@CZMA-LDH_R*y*, where *y* = 1, 2, 3, 4 or 5, depending of cycle number) to obtain pure LDH material with high metal incorporation. A salt solution concentration of 15 mM was used and 1 g of the solid sample was mixed with 60 mL of the solution. After five cycles, the theoretical Cu^2+^ and Zn^2+^ content in the sample is similar to that of H-S@CZMA-LDH_20 mM obtained from a single immersion approach in 20 mM solution (4.5 *vs.* 4.0 mmol_Cu+Zn_ g^−1^).

The elemental composition of the samples after each calcination–reconstruction cycle was analysed by ICP-OES as shown in Fig. S9.† The (Cu + Zn)/Al molar ratio in the reconstructed samples steadily increases with each cycle, reaching 0.18, 0.36, 0.53, 0.74 and 0.96, while the Mg/Al ratio decreases from 2.28 to 2.11, 1.98, 1.78, 1.57 and 1.25. These trends suggest a stepwise exchange of divalent metal ions during the calcination–reconstruction cycle. In addition, the ratio of (Cu + Zn + Mg)/Al remained relatively constant (2.21–2.34), regardless of the number of cycles. Given the initial Mg/Al of 2.28, the exchange ratio between Mg^2+^ and Cu^2+^ + Zn^2+^ appears to be 1 to 1, which is the same as that found in the single immersion approach. The Cu/Zn ratio across all sample was 1.42–1.44, closely matching the theoretical value of 1.30. Based on these results, the metal composition of the LDH in H-S@CZMA-LDHs after successive cycles is determined as follows: Cu_0.10_Zn_0.07_Mg_2.11_Al, Cu_0.21_Zn_0.15_Mg_1.98_Al, Cu_0.31_Zn_0.22_Mg_1.78_Al, Cu_0.44_Zn_0.31_Mg_1.57_Al_1.00_, Cu_0.56_Zn_0.39_Mg_1.25_Al, respectively.

**Fig. 5:**
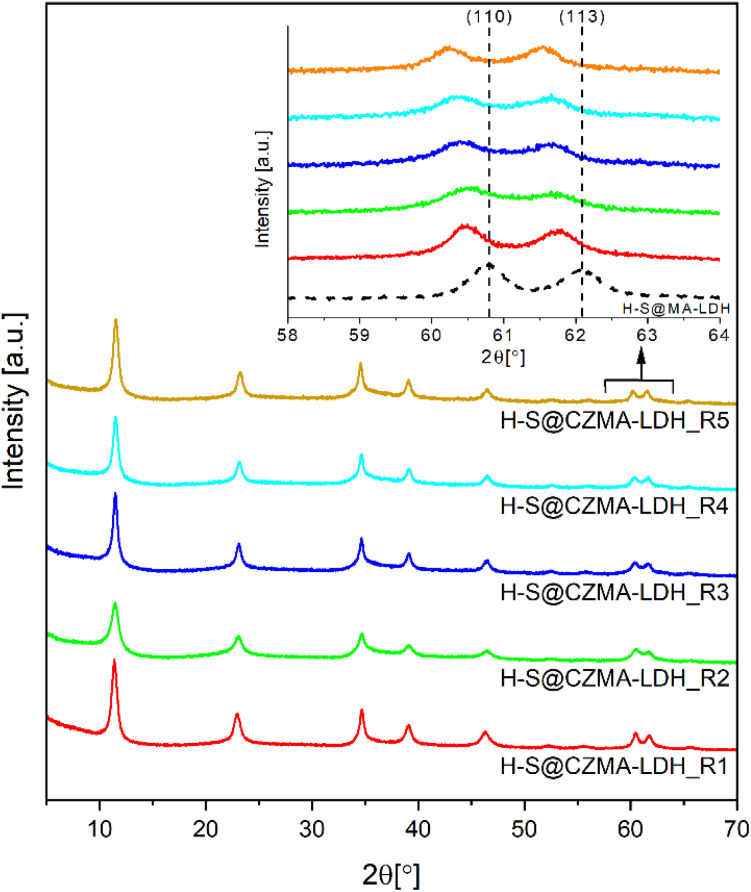
XRD patterns for H-S@CZMA-LDHs after five successive iterations of calcination–reconstruction with a 15 mM CuZn-containing solution.

The XRD patterns of the samples after each cycle are shown Fig. 5. In all cases, only distinct characteristic Bragg reflections for a crystalline LDH structure are observed. Notably, a systematic shift in the positions of (110) and (113) Bragg reflections toward lower 2*θ* values is observed as highlighted in the inset of Fig. 5. This shift is attributed to the differing ionic radii of Mg^2+^ compared to the Cu^2+^ and Zn^2+^ ions that replace it (octahedral ionic radii: Mg^2+^ 0.72 Å, Cu^2+^ 0.73 Å and Zn^2+^ 0.74 Å).^52^ The substitution affects the lattice parameters (Table 1). Particularly, the *a*-lattice parameter (the metal–metal distance in brucite-like layers) increases gradually from 0.3049 nm in the parent H-S@MA-LDH to 0.3073 nm after five cycles. This increase reflects the progressive replacement of Mg^2+^ ions with Cu^2+^ and Zn^2+^ ions, consistent with the ICP-OES analysis in Fig. S9.† The *c*-lattice parameter remains within 2.302–2.318 nm, corresponding to an LDH basal spacing of 7.67–7.73 Å with CO_3_^2−^ ions in the interlayer space.^53^ The presence of CO_3_^2−^ anions in the interlayer space were further confirmed by IR spectroscopy (Fig. S10†). All reconstructed samples, regardless of the number of the calcination–reconstruction cycles, possess a similar spectral shape with a clearly distinct *ν*(C–O) stretching mode at 1360 cm^−1^.

**Table 1:** Crystal and textural properties of the H-S@MgAl-LDH and H-S@CZMA-LDHs after five successive iterations of calcination–reconstruction with a 15 mM CuZn-containing solution

Sample	LDH lattice parameters^a^ [nm]		LDH *d*(003) [nm]	LDH crystallite size^b^ [nm]		*S*_BET_ [m^2^ g^−1^]	*V*_micro_ [cm^3^ g^−1^]	*V*_meso_ [cm^3^ g^−1^]	*V*_total_ [cm^3^ g^−1^]
	*a*	*c*		*D*_(003)_	*D*_(110)_				
H-S@MA-LDH	0.3049	2.308	0.769	7.9	17.9	224	0.008	0.66	0.89
H-S@CZMA-LDH_R1	0.3062	2.318	0.773	13.9	15.0	163	0.009	0.68	0.67
H-S@CZMA-LDH_R2	0.3061	2.318	0.773	9.8	9.2	157	0.012	0.70	0.66
H-S@CZMA-LDH_R3	0.3065	2.312	0.771	15.8	9.9	158	0.012	0.74	0.73
H-S@CZMA-LDH_R4	0.3067	2.306	0.769	14.8	10.9	158	0.010	0.75	0.74
H-S@CZMA-LDH_R5	0.3073	2.302	0.767	14.2	13.5	179	0.002	0.66	0.74

a *a* = 2*d*_110_, *c* = 3*d*_003_.

b Crystallite size of LDH in stacking or plane direction (*D*_(003)_ and *D*_(110)_, respectively), calculated according to Scherrer equation: *D*_(*hkl*)_ = 0.9 × λ/(*β* × cos *θ*), *λ* = 0.154 nm, *θ* is the position (deg.), and *β* is the FWHM (rad.) of the Bragg diffraction peaks.

The H-S@CZMA-LDHs exhibited similar porosity with nearly identical adsorption–desorption type IVa isotherms and H3 hysteresis loops (Fig. S11†). Their specific BET surface area and pore volumes are summarised in Table 1. The *S*_BET_ and *V*_total_ are in the range of 158–179 m^2^ g^−1^ and 0.66–0.74 cm^3^ g^−1^, respectively. The mesopore volumes are between 0.68–0.75 cm^3^ g^−1^, with the highest volume obtained after the fourth reconstruction cycle. Although, no clear correlation was found between porosity and the number of modifications, the calcination–reconstruction process had a minimal impact on the overall porosity of the materials.

### Spherical hollow-SiO_2_@Cu_*x*_Zn_*y*_Mg_*z*_Al as catalyst precursors

To deconvolute the relationship between catalyst structure/composition (including copper phase and size) and catalytic CO_2_-to-methanol activity, three samples (H-S@CZMA-LDH_20 mM, H-S@CZMA-LDH_80 mM and H-S@CZMA-LDH_R5) were selected as promising catalyst precursors. As shown by elemental analysis (Table S5†), H-S@CZMA-LDH_80 mM possesses two times the Cu + Zn content of H-S@CZMAl-LDH_20 mM but contains two phases of Cu_2_(OH)_3_NO_3_ and CuZnMgAl-LDH. In turn, H-S@CZMA-LDH_R5, developed after five calcination–reconstruction cycles, retains a pure LDH structure with a similar metal content to H-S@CZMAl-LDH_20 mM. All samples were calcined in air at 330 °C and reduced in H_2_ at 290 °C.

The relationship between CO_2_ conversion, selectivity to methanol and CO, and reaction temperature is detailed in Fig. S12A–C.† All catalysts exhibit an increase in CO_2_ conversion and CO selectivity and a decrease in methanol selectivity with increasing reaction temperature. This is consistent with thermodynamic control for CO_2_ hydrogenation.^5^ CO is the main by-product generated *via* a reverse water gas shift process (RWGS, CO_2_ + H_2_ ⇌ CO + H_2_O) which is an endothermic reaction (Δ*H*^0^ = 41.2 kJ mol^−1^). When the temperature increases, the RWGS becomes more predominant, leading to an increased CO selectivity and a decreased methanol selectivity. The catalysts with comparable Cu + Zn loading (H-S@CZMA_20 mM and H-S@CZMA_R5) show comparable CO_2_ conversion, reaching 16.5 and 17.5% at 290 °C, respectively. In contrast, the Cu-rich catalyst (H-S@CZMA_80 mM) shows poor activity for CO_2_ hydrogenation with a CO_2_ conversion rate below 2.4%. As the temperature increases, MeOH selectivity drops from 70.6 to 21.3% and from 65.4 to 19.7% for H-S@CZMA_20 mM and H-S@CZMA_R5, respectively. However, H-S@CZMA_80 mM exhibits a more moderate decrease in methanol selectivity (from 85.2 to 58.2%) over the same temperature range.

**Fig. 6:**
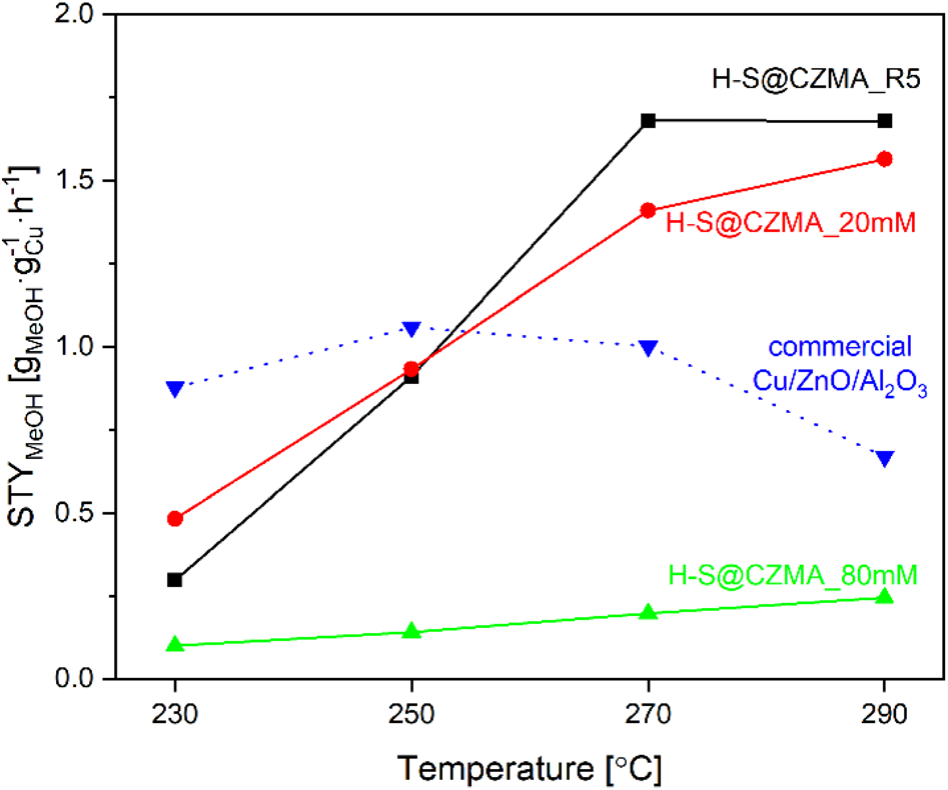
The space-time yield for methanol production (STY_MeOH_) of H-S@CZMA catalysts and commercial Cu/ZnO/Al_2_O_3_ as a function of temperature (CO_2_ : H_2_ = 3, 30 bar).

Given the wide range of reaction conditions used by different research groups, a direct comparison of the catalytic performance of our catalysts with those reported in the literature is challenging. However, we have summarised the performance of various Cu-containing catalysts with respect to the reaction conditions in Table S6.† We have been able to compare the catalytic efficiency of our catalysts with the commercial Cu/ZnO/Al_2_O_3_ catalyst, under the identical reaction conditions (Fig. S12D†). Although on a gram catalyst basis, the commercial Cu/ZnO/Al_2_O_3_ catalyst showed better CO_2_ hydrogenation efficiency to methanol with CO_2_ conversion of 9.4–22.2% and selectivity of 52.9–17.1% to MeOH in the temperature range of 230–290 °C, the situation changes dramatically when we normalise on a per Cu wt% basis. After normalisation, the space-time yield of methanol (STY_MeOH_) per gram Cu (g_Cu_) is shown in Fig. 6. At low temperature (230 °C), the commercial Cu/ZnO/Al_2_O_3_ catalyst is more effective than our catalysts. However, at higher temperatures, H-S@CZMA_20 mM and H-S@CuZnMgAl_R5 showed significantly higher STY_MeOH_. At 270 °C, the STY_MeOH_ for H-S@CZMA_20 mM and H-S@CZMA_R5 are 1.4 and 1.7 times higher than the commercial Cu/ZnO/Al_2_O_3_ catalyst (1.41 and 1.68 *vs.* 1.00 g_MeOH_ g_Cu_^−1^ h^−1^, respectively). This advantage became even more pronounced at the higher temperature, where determined values of STY_MeOH_ was 1.56 and 1.68 *versus* 0.67 g_MeOH_ g_Cu_^−1^ h^−1^, respectively.

In order to understand the correlation between catalyst structure and catalytic CO_2_-to-methanol activity, comprehensive characterisation studies were conducted on the synthesised catalysts. XRD measurements were performed for thermally activated samples. After calcination at 330 °C (Fig. S13A†), the H-S@CZMA-LDH_20 mM and H-S@CZMA-LDH_R5 converted into equivalent amorphous LDO solids, with the crystalline LDH features disappearing due to dehydration, dehydroxylation and partially decarbonylation. Two broad scattering features are observed at *ca.* 2*θ* = 35.3 and 61.0° which are attributed to the formation of poorly-crystalline ZnO phase.^54^ In contrast, the H-S@CZMA-LDO_80 mM showed highly the presence of a crystalline CuO phase, most likely due to its high Cu content (approx. 39 at%) of Cu_2_(OH)_3_NO_3_ in H-S@CZMAl-LDH_80 mM, which facilitated CuO formation.^47^ After reduction in H_2_ at 290 °C (Fig. S13B†), an additional signal at 2*θ* = 43.3° appears in both H-S@CZMA_20 mM and H-S@CZMA_R5. This weak scattering feature is assigned to the (111) Bragg reflection of metallic nanocopper (Cu^0^). H-S@CZMA_80 mM presented two additional sharp Bragg reflections at 2*θ* = 43.4° and 50.6°, which could be assigned to the (111) and (200) Bragg reflections of Cu^0^.^55^ These differences in crystallinity of the metallic Cu phases in the catalysts were quantified using the Scherer equation. For H-S@CZMA_20 mM and H-S@CZMA_R5, the mean Cu crystallite domain lengths are 3.2 and 2.7 nm, respectively. While the highly crystalline H-S@CZMA_80 mM possesses Cu crystallites with much larger domain lengths of 33.9 nm.

TEM electron microscopy was used to evaluate the size distribution of metallic copper particles in our samples, as shown in Fig. S14.† The H-S@CZMA_20 mM sample presents a narrow Cu particle size distribution (2–6 nm) with an average size of 3.8 ± 0.7 nm. Similarly, the H-S@CZMA_R5 catalyst showed Cu nanoparticles predominantly within the same range, with a slightly broader average size of 3.6 ± 1.2 nm. In contrast, H-S@CZMA_80 mM catalyst displays significantly larger Cu particles, with 66% of the particles in the 10–40 nm range and an average size of 30.1 ± 18.5 nm.

These insights into the Cu particle size, supported by the results of XRD and TEM measurements, allow us to conclude that this parameter is strongly dependent on the conditions applied during the reconstruction process. Both of the more active methanol catalysts contains similarly small sized metallic copper nanoparticles incorporated into the catalyst precursor matrix (pure LDH) using less concentrated solutions, regardless of whether a single immersion or multiple calcination–reconstruction cycles were used. Conversely, the use of highly concentrated copper solution in single immersion process resulted in the formation of large particles/aggregates of metallic copper, leading to materials with the low catalytic efficiency.

## Conclusions

We have developed a systematic synthesis approach to hollow-SiO_2_@Cu_*x*_Zn_*y*_Mg_*z*_Al-LDHs where LDH nanosheets are anchored perpendicularly to the surface of thin SiO_2_ shells. Our approach leverages the memory effect of LDHs to regenerate hollow-SiO_2_@Cu_*x*_Zn_*y*_Mg_*z*_Al-LDHs within a pre-formed hollow-SiO_2_@Mg_2_Al-LDH template. We have demonstrated that the concentration of Cu^2+^ and Zn^2+^ solution significantly influences the composition, crystallinity, and porosity of the reconstructed LDH. A pure Cu_*x*_Zn_*y*_Mg_*z*_Al-LDH phase with desired morphology and porosity can be obtained in a single immersion approach using 20 mM. Higher concentration solutions greater than 40 mM result in the poorly crystalline LDH phase and a new phase with high crystallinity (Cu_2_(OH)_3_NO_3_), leading to lower porosity and poorer hollow morphology. Another approach using multiple calcination–reconstruction cycles allows gradual incorporation of Cu^2+^ and Zn^2+^ ions into the pure and crystalline LDH matrix, while maintaining good morphology and high porosity. Furthermore, our investigation underscored the significant influence of the reconstruction conditions on the formation of catalytically active phase. It was clearly demonstrated that a catalyst with a high content of the Cu^0^ metallic phase, but in the form of large particles, shows a significantly lower efficiency in methanol production compared to those with a lower Cu content, but smaller and well-dispersed Cu^0^ particle sizes. We believed that this research provides valuable insight into the design and synthesis of a new generation of hollow spherical material with promising properties and potential, particularly in fields related to climate protection and CO_2_ management.

## Data availability

General experimental details, synthetic protocols and additional characterising data supporting this article have been included as part of the ESI.†

## Author contributions

T. Kondratowicz performed the synthetic and experimental work as well as conceptualised the research; M. Gajewska assisted with the electron microscopy studies; M. Li and J. Li conducted the catalytic CO_2_ to MeOH experiments; C. Chen and Z. R. Turner supervised the work; D. O'Hare conceptualised and supervised the research and acquired the funding.

## Conflicts of interest

There are no conflicts to declare.

## Supplementary Material

SC-OLF-D4SC07292H-s001
