# Supplementary material for: Hollow-SiO2@CuxZnyMgzAl-LDHs as catalyst precursors for CO2 hydrogenation to methanol
Source: Chem Sci. 2024 Dec 17;16(3):1327–35. doi: 10.1039/d4sc07292h (PMC11651139; doi:10.1039/d4sc07292h)
Supplement: SC-016-D4SC07292H-s001 [file SC-016-D4SC07292H-s001.pdf]

Supplementary Information (SI) for Chemical Science.
This journal is © The Royal Society of Chemistry 2024

Supplementary material

Hollow-SiO_2@$Cu_xZn_yMg_zAl$-LDHs as catalyst precursors for CO_2 hydrogenation to methanol

Tomasz Kondratowicz,[a] Marta Gajewska,[b] Jiangtong Li,[c] Molly Meng-Jung Li,[c] Zoë R. Turner,[a] Chunping Chen*[a] and Dermot O'Hare*[a]

[a] Chemistry Research Laboratory, Department of Chemistry, University of Oxford, 12 Mansfield Road, Oxford, OX1 3TA, UK. E-mail: chunping.chen@chem.ox.ac.uk; dermot.ohare@chem.ox.ac.uk; Tel: +44(0)1865 272686

[b] Academic Centre for Materials and Nanotechnology, AGH University of Krakow, Mickiewicza 30, 30-059 Krakow, Poland

[c] Department of Applied Physics, The Hong Kong Polytechnic University, P. R. China

Contents

1. Experimental section
2. Supporting figures
3. Supporting tables

Experimental section

Synthesis

Solid SiO_2@Mg_2Al-LDH core-shell

The spherical solid SiO_2@Mg_2Al-LDH core-shell (with Mg/Al molar ratio = 2) were prepared via in-situ coprecipitation of Mg and Al precursor in the presence of non-porous spherical SiO_2 particles with uniform size with the diameters ca. 350 nm, according to our previous work.[1] Briefly, 100 mg of spherical SiO_2 particles were dispersed in 20.0 mL of deionized water using ultrasound bath. After 1 h, 0.96 mmol of Na_2CO_3 (Sigma-Aldrich, 99.5%) was added to the milky solution and dispersed for another 10 minutes. Subsequently, to so prepared suspension added dropwise (1 $mL{\cdot}min^{-1}$) 19.2 mL water solution containing 0.96 mmol $Mg(NO_3)_2{\cdot}6H_2O$ (Sigma-Aldrich, 99%) and 0.48 mmol $Al(NO_3)_3{\cdot}9H_2O$ (Sigma-Aldrich, 98%). The pH was maintained at 10.00 ± 0.03 using an aqueous NaOH solution (1 M) during titration. After 2 h of stirring, the obtained core-shell particles were filtered, washed with deionized water and dried under vacuum at 30 °C overnight. Dried sample was denoted as S@MA-LDH.

Hollow-SiO_2@Mg_2Al-LDH spheres

The silica core from solid SiO_2@Mg_2Al-LDH was selectively etched under static conditions by treatment with a NaOH solution (1 M) at 30 °C for 4 h. To remove remaining NaOH, the obtained particles were thoroughly washed with distilled water and then dried under vacuum at 30 °C overnight. Dried sample was denoted as H-S@MA-LDH.

Hollow-SiO_2@Mg_2Al-LDO spheres

Hollow-SiO_2@Mg_2Al-LDH precursor was calcined at 450 °C for 3 h (heating rate of 5 $°C{\cdot}min^{-1}$) in a muffle oven under static air. Calcined material was denoted as H-S@MA-LDO.

Hollow-SiO_2@$Cu_xZn_yMg_zAl$-LDH spheres synthesised via memory effect

First, binary aqueous solutions containing Cu^{2+} and Zn^{2+} cations at total concentrations of 15, 20, 40, 60 and 80 mM (molar ratio of Cu to Zn = 1.30) were prepared by dissolving the appropriate mass of $Cu(NO_3)_2{\cdot}3H_2O$ (Supelco, 99.5%) and $Zn(NO_3)_2{\cdot}6H_2O$ (Alfa Aesar, 99%) in deionized water.

Typically, for a single reconstruction, 1.00 g of H-S@MA-LDO was mixed with 200 mL of an aqueous solution containing Cu^{2+} and Zn^{2+} ions at a stirring speed of 250 rpm at room temperature. After 24 hours, the solid samples were isolated by filtration, washed and dried under vacuum at 30 °C overnight. For this modification, solutions containing Cu^{2+} and Zn^{2+} cations at total concentrations of 20, 40, 60 and 80 mM were used. The rehydrated samples were designated as H-S@CZMA-LDH_*x*mM, where *x* is the concentration of the solution used.

The repetition of the calcination-reconstruction experiments was studied with 15 mM CuZn-containing solution, and the ratio of the volume of the solution to the mass of the calcined solid was equal 60 mL·1.00 g^{-1}. After each immersion, the rehydrated solid sample was washed and dried, and before the following reconstruction process was calcined in a muffle furnace in air at 450 °C for 3 h (at a heating rate of 5 °C·min^{-1}). Five such repetitions were performed, and the samples were designated as H-S@CZMA-LDH_R*y*, where *y* = 1, 2, 3, 4 or 5, depending of cycle number.

Characterization

Transmission electron microscopy (TEM) measurements were carried out on a FEI Tecnai TF20 X-TWIN (FEG) microscope equipped with an energy-dispersive X-ray spectrometer (EDAX), working at an accelerating voltage of 200 kV. Samples for the TEM observations were prepared by drop-casting on carbon-coated copper grids. The average particle diameter of core-shell particles and metallic copper particles in studied catalysts was obtained by analysis of ca. 200 counting and using the arithmetic mean and standard deviation functions.

Crystallographic structure of samples was determined by Bruker D8 diffractometer with CuKα radiation (λ_1 = 1.544 Å and λ_2 = 1.541 Å) operated at 40 kV and 30mA. Solid samples were mounted on PMMA sample holders and XRD patterns were recorded in the range of 2θ = 5-70° with a step of 0.01°.

Nitrogen adsorption-desorption isotherms were recorded at -196 °C using a Micromeritics TriStar II instrument. Before the measurements, samples were degassed overnight under vacuum at 130 °C or 150 °C for LDH and LDO phase, respectively. Specific surface areas (S_{BET}) were determined by the BET method. Total pore volumes (V_{total}) were obtained from amounts of nitrogen adsorbed at relative pressure of 0.99, whereas micropore (V_{micro}) and mesopore (V_{meso}) volumes were calculated using the t-plot and Barrett-Joyner-Halenda (BJH) models, respectively.

Inductively coupled plasma optical emission spectroscopy (ICP-OES) analyses were carried out at Department of Chemistry, University of Cambridge. Measurements were performed on a Themo Fisher Scientific iCAP 7400 Duo ICP Spectrometer. Calibration was performed by construction of a standard curve using ICP standards from Sigma-Aldrich. Samples were run in ~2 % nitric acid (Fisher TraceMetal grade). Samples were weighed on a Mettler UMT2 balance and dissolved in 5 mL nitric acid, diluted with 5 mL water and a 0.5 mL aliquot was diluted to 10 mL with water.

Elemental analysis was performed using a ThermoFlash 2000 (Thermo Scientific) CHN analyser at London Metropolitan University.

Thermogravimetric analyses (TGA) were performed using a PerkinElmer TGA 8000. A sample of approximately 10 mg was heated under a nitrogen atmosphere (20 mL·min^{-1}) from 30 to 800 °C at a heating rate of 5 °C·min^{-1}.

FT-IR spectra were measured on a Bruker Vertex 80 spectrometer fitted with a DuraSamplIR Diamond ATR accessory. 256 background scans were taken before 128 sample scans were recorded, capturing the transmittance signals between 400-4000 cm^{-1} at a 2 cm^{-1} resolution.

Catalytic tests

All precursor materials were subjected to calcination under a constant air flow of 50 mL·min^{-1} at 330 °C for 3 h (heating rate of 10 °C·min^{-1}) followed by 1 hour of dwell at 80 °C and 150 °C) prior to use. The CO_2 hydrogenation reactions were conducted in a high-pressure fixed-bed continuous-flow reactor, which was equipped with online gas chromatography (GC) for product analysis. For the catalytic tests, 0.1 g of the calcined catalyst precursor was diluted with 0.2 g of quartz sand (20-30 mesh) and placed in the catalyst bed. Prior to the reaction, the calcined catalyst precursor was reduced at 290 °C for 2 h using pure H_2 at a flow rate of 20 mL·min^{-1} (standard temperature and pressure, stp; P = 101.3 kPa, T = 298 K) under atmospheric pressure. Following reduction, the reactor temperature was cooled to 50 °C, and the reactor was subsequently pressurized to 3.0 MPa using a reactant gas mixture of $CO_2/H_2/N_2$ in a molar ratio of 24:72:4, with N_2 serving as the internal standard. Activity measurements were performed under a constant flow of reactant gas (30 mL·min^{-1}, stp) through the catalyst bed at temperatures of 230 - 290 °C. Measurements were taken after maintaining the reaction conditions for at least 2 hours at each specified temperature. The product gases exiting the reactor were maintained at 130 °C and immediately transported to the SHIMADZU GC-2030 gas chromatograph. The GC was equipped with two thermal conductivity detectors (TCD) and a flame ionization detector (FID) for quantitative analysis.

Supporting figures

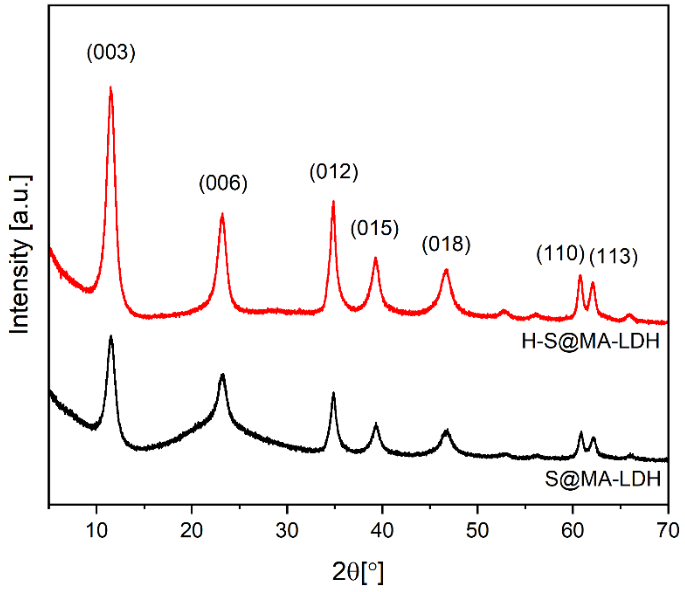

Figure S1. XRD patterns of S@MA-LDH core-shell (black) and H-S@MA-LDH spheres (red).

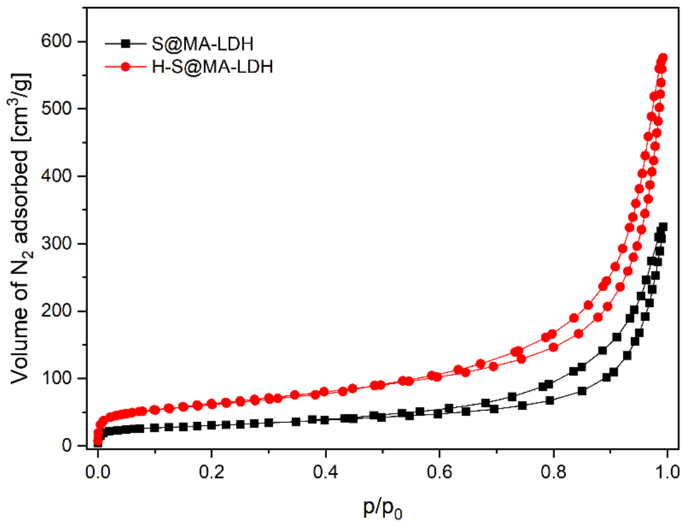

Figure S2. N_2 adsorption and desorption isotherm of S@MA-LDH (black) and H-S@MA-LDH (red).

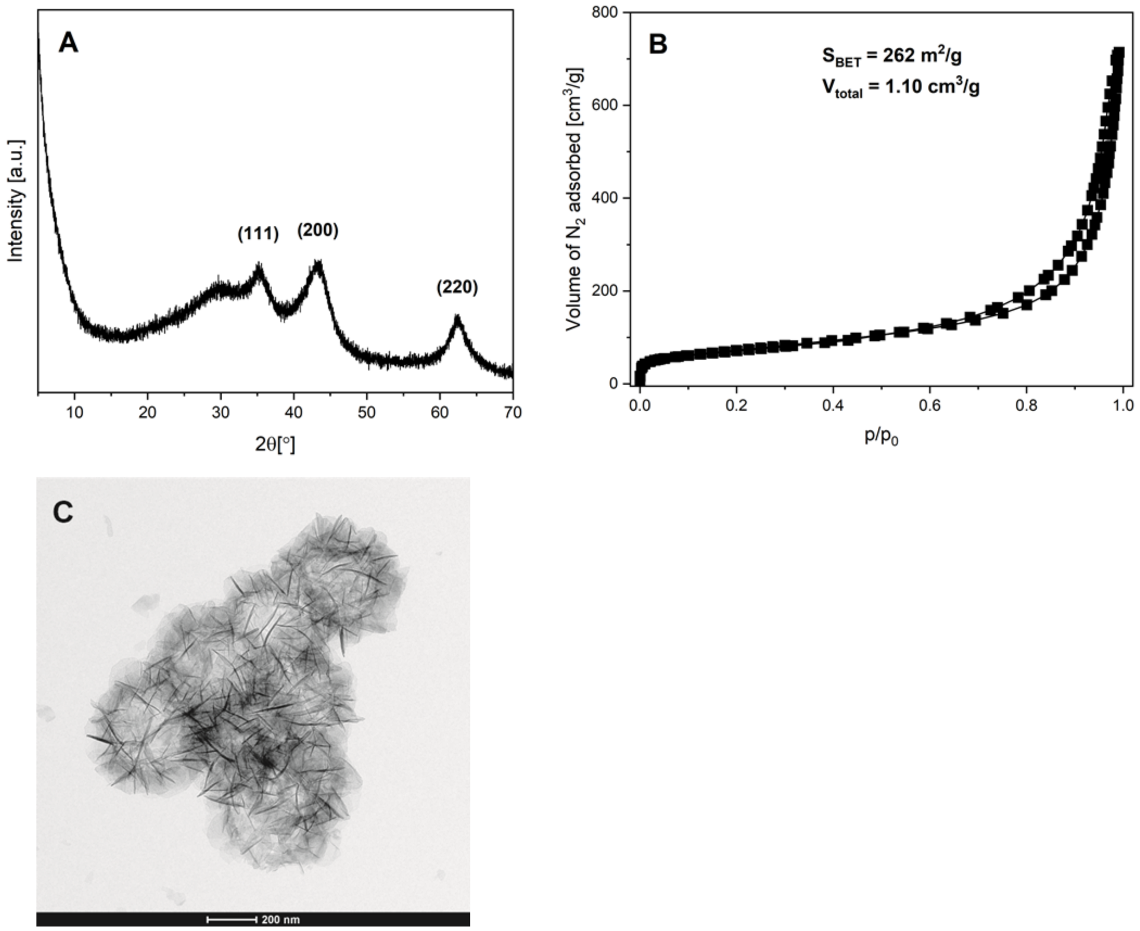

Figure S3. XRD pattern (A), N_2 adsorption-desorption isotherm (B) and TEM image (C) of H-S@MA-LDO after calcination at 450 °C.

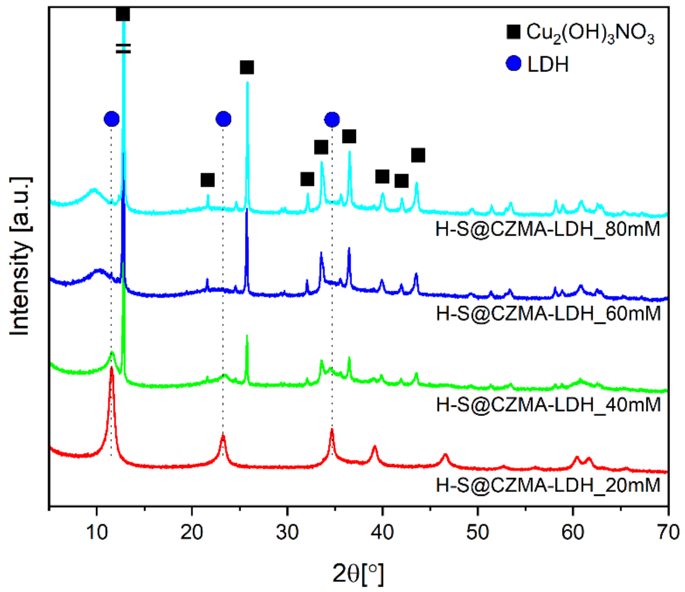

Figure S4. XRD patterns for H-S@CZMA-LDHs after immersion in various Cu^{2+}+Zn^{2+}-containing solutions (20-80 mM).

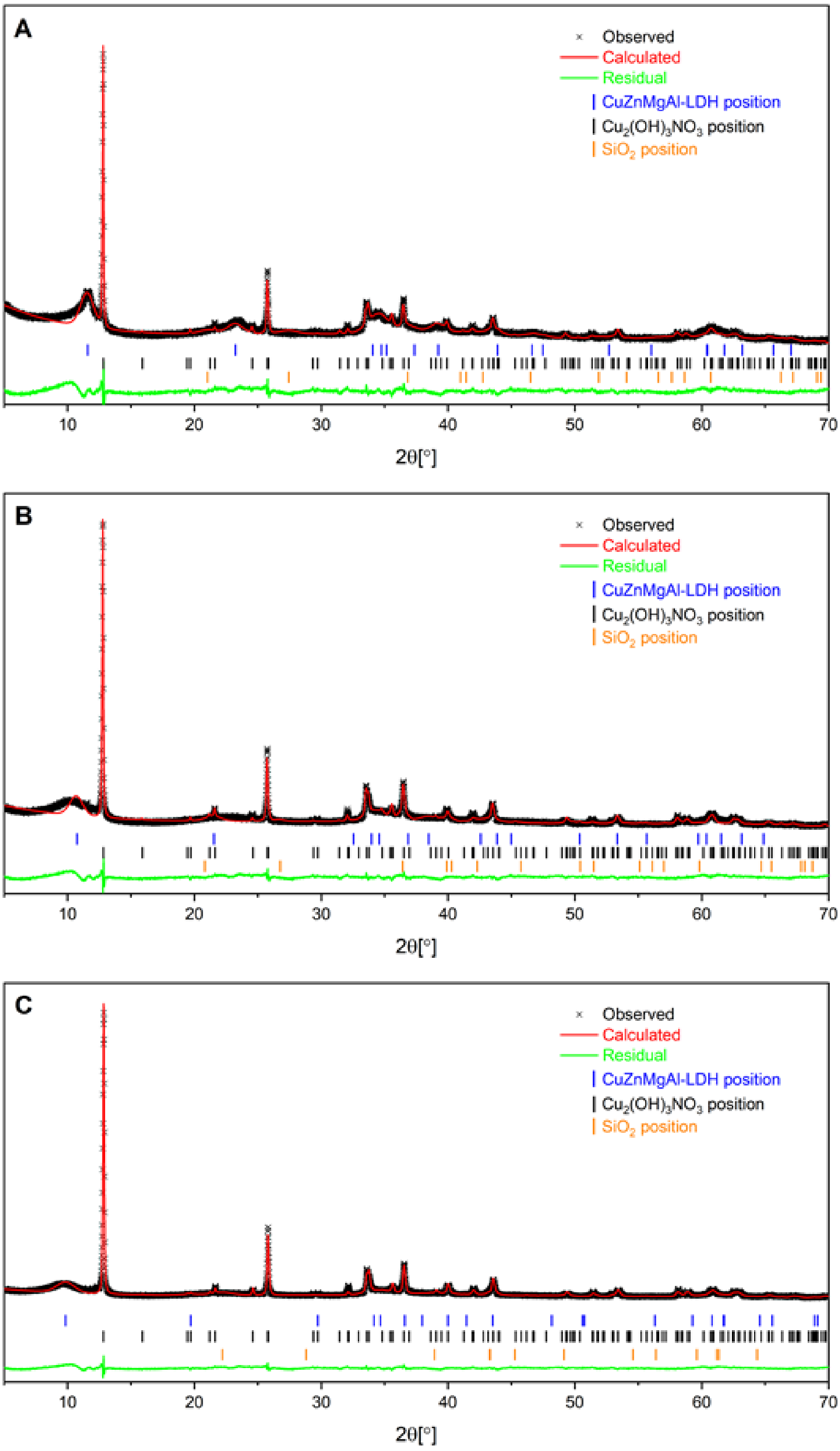

Figure S5. XRD patterns and Rietveld refinement for H-S@CZMA-LDHs after immersion in 40 mM (A), 60 mM (B) and 80 mM (C) solutions (total concentration of Cu^{2+} and Zn^{2+}). The observed, calculated, background and difference are shown.

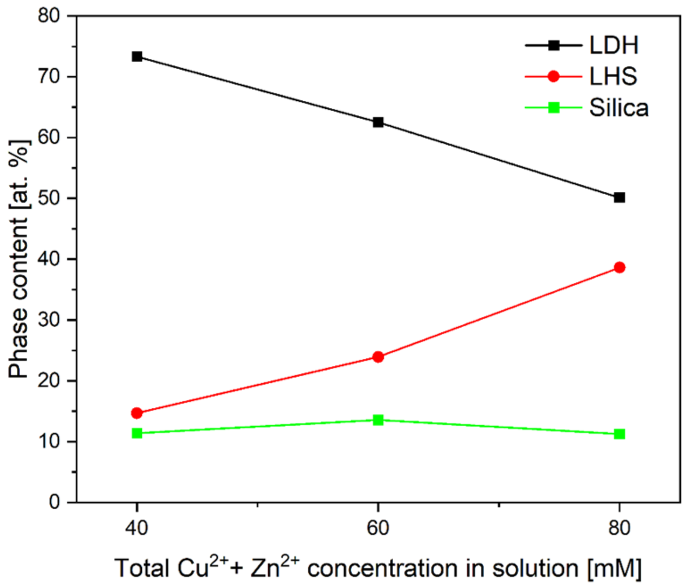

Figure S6. Phase composition of H-S@CZMA-LDHs depending on the initial concentration of CuZn-containing solutions, determined by Rietveld refinement.

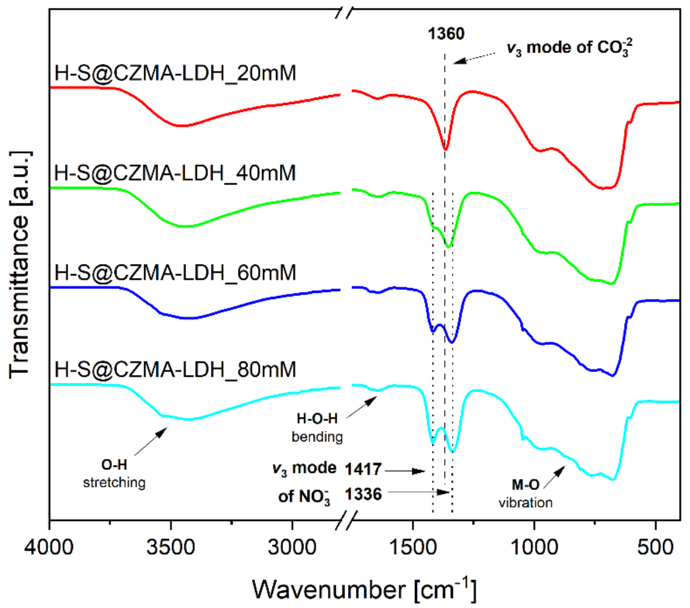

Figure S7. FT-IR spectra of the H-S@CZMAl-LDHs after reconstruction with various CuZn-containing solutions (20-80 mM in total).

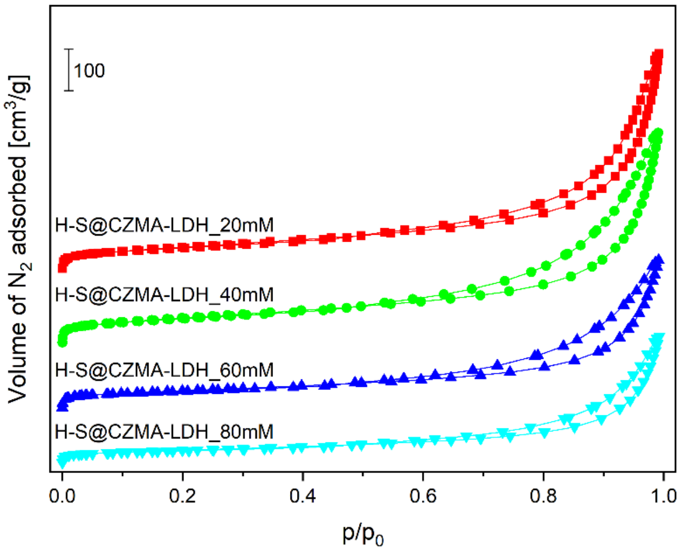

Figure S8. N_2 adsorption-desorption isotherms of the H-S@CZMA-LDHs after reconstruction with various Cu^{2+}+Zn^{2+}-containing solutions (20-80 mM).

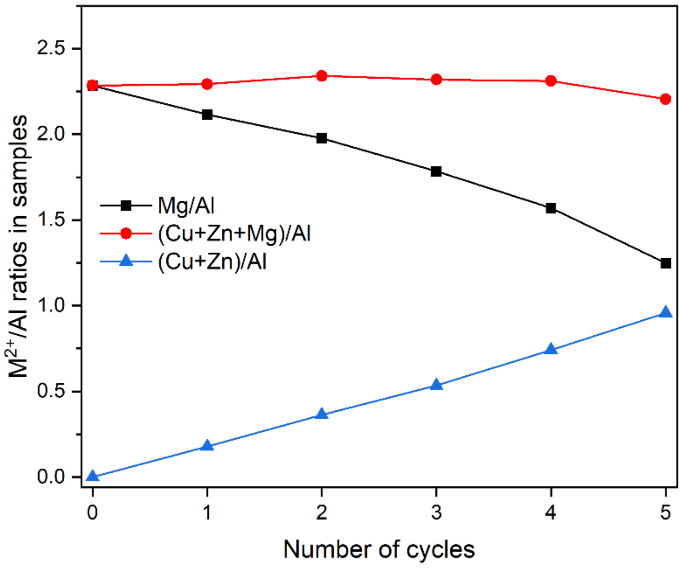

Figure S9. Ratios of Mg/Al, (Cu+Zn+Mg)/Al and (Cu+Zn)/Al in H-S@CZMA-LDHs depending on the on the number of calcination-reconstruction cycles.

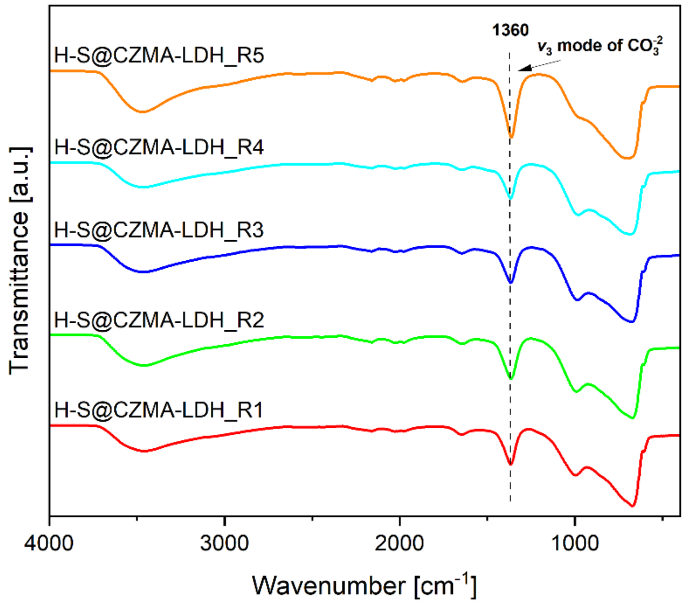

Figure S10. FT-IR spectrums for H-S@CZMA-LDHs after five successive iterations of calcination- reconstruction with a 15 mM CuZn-containing solution.

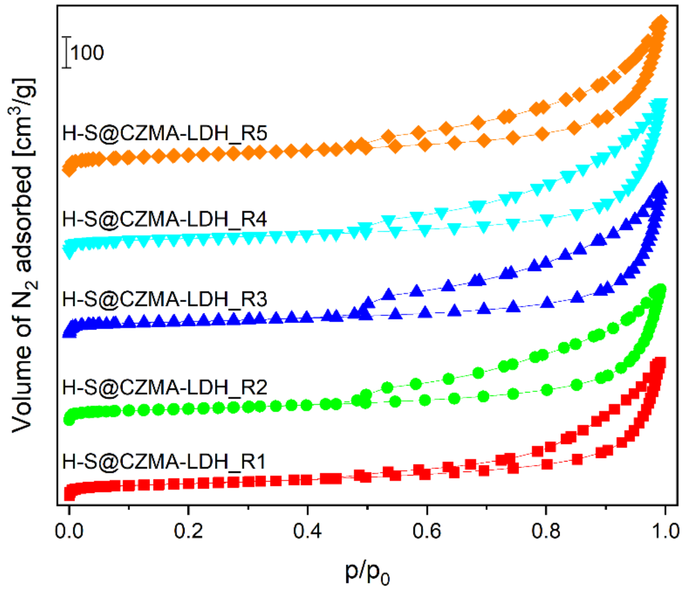

Figure S11. N_2 adsorption-desorption isotherms of the H-S@CZMA-LDHs after five successive iterations of calcination-reconstruction with a 15 mM CuZn-containing solution.

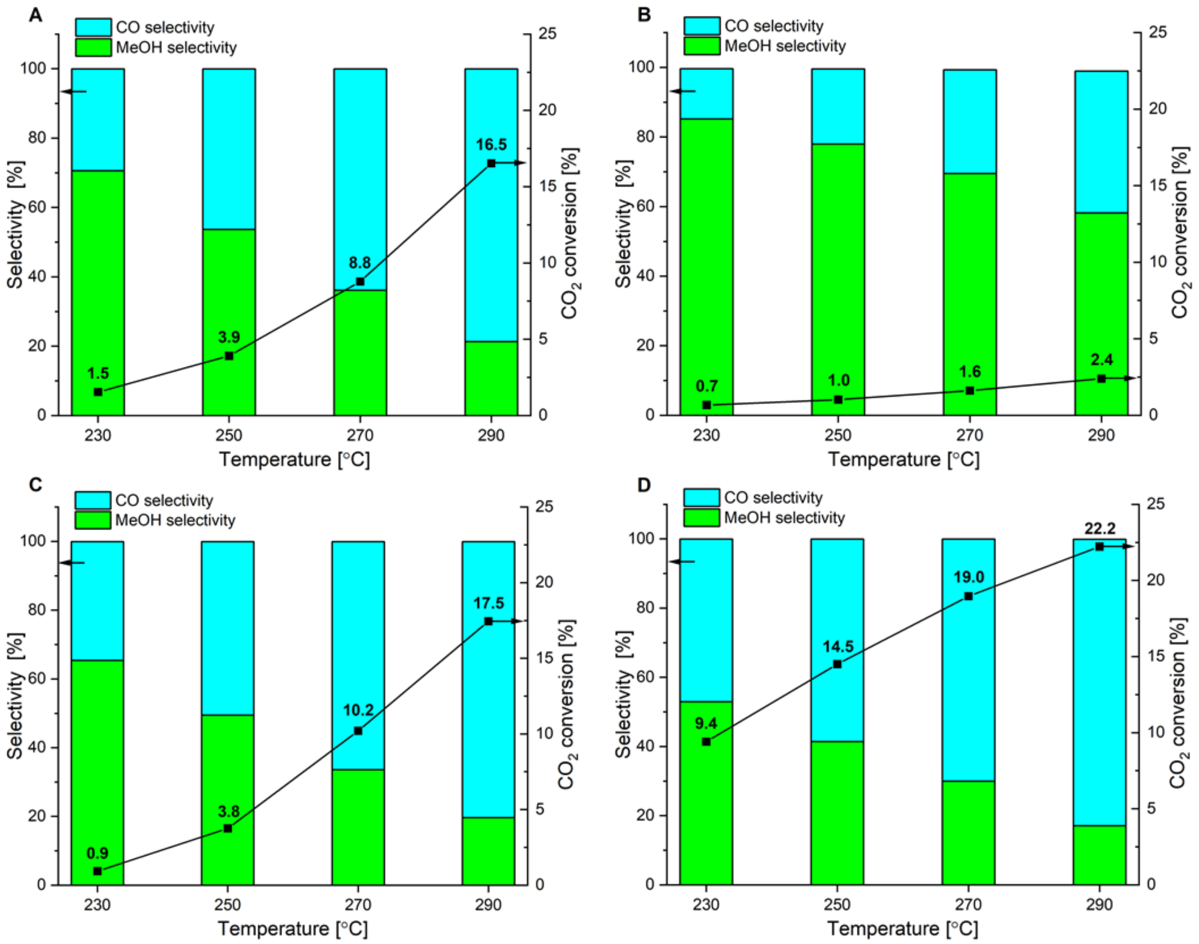

Figure S12. Catalytic performance of H-S@CZMA_20mM (A), H-S@CZMA_80mM (B), H-S@CZMA_R5 (C) and commercial Cu/ZnO/Al_2O_3 (D) for CO_2 hydrogenation to methanol.

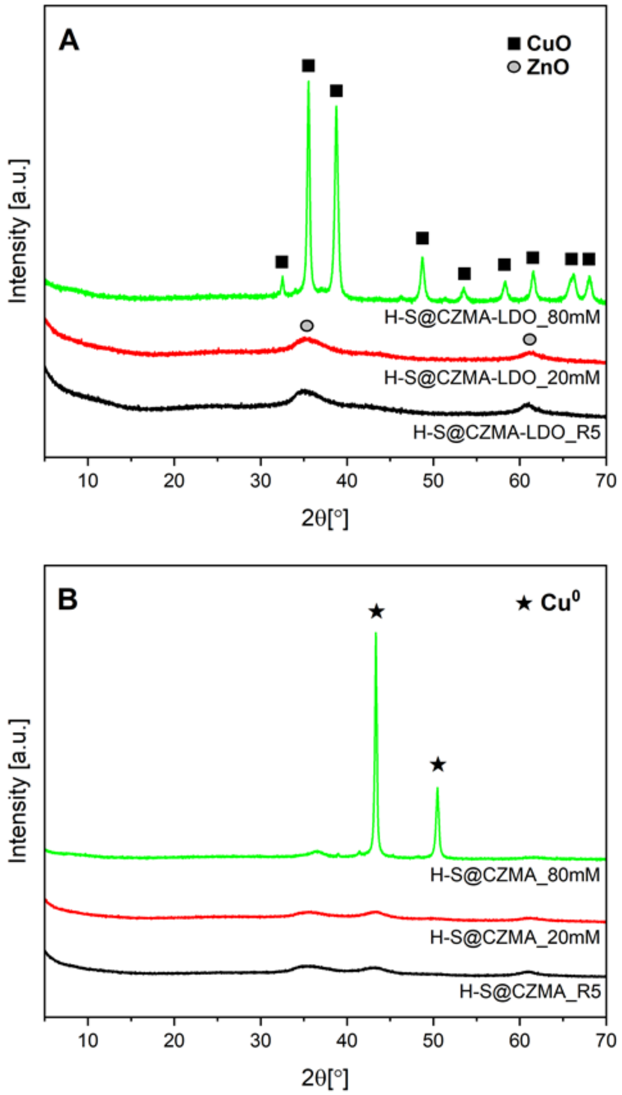

Figure S13. XRD profiles of studied samples after calcination in air at 330 °C (A) and reduction in H_2 at 290 °C (B).

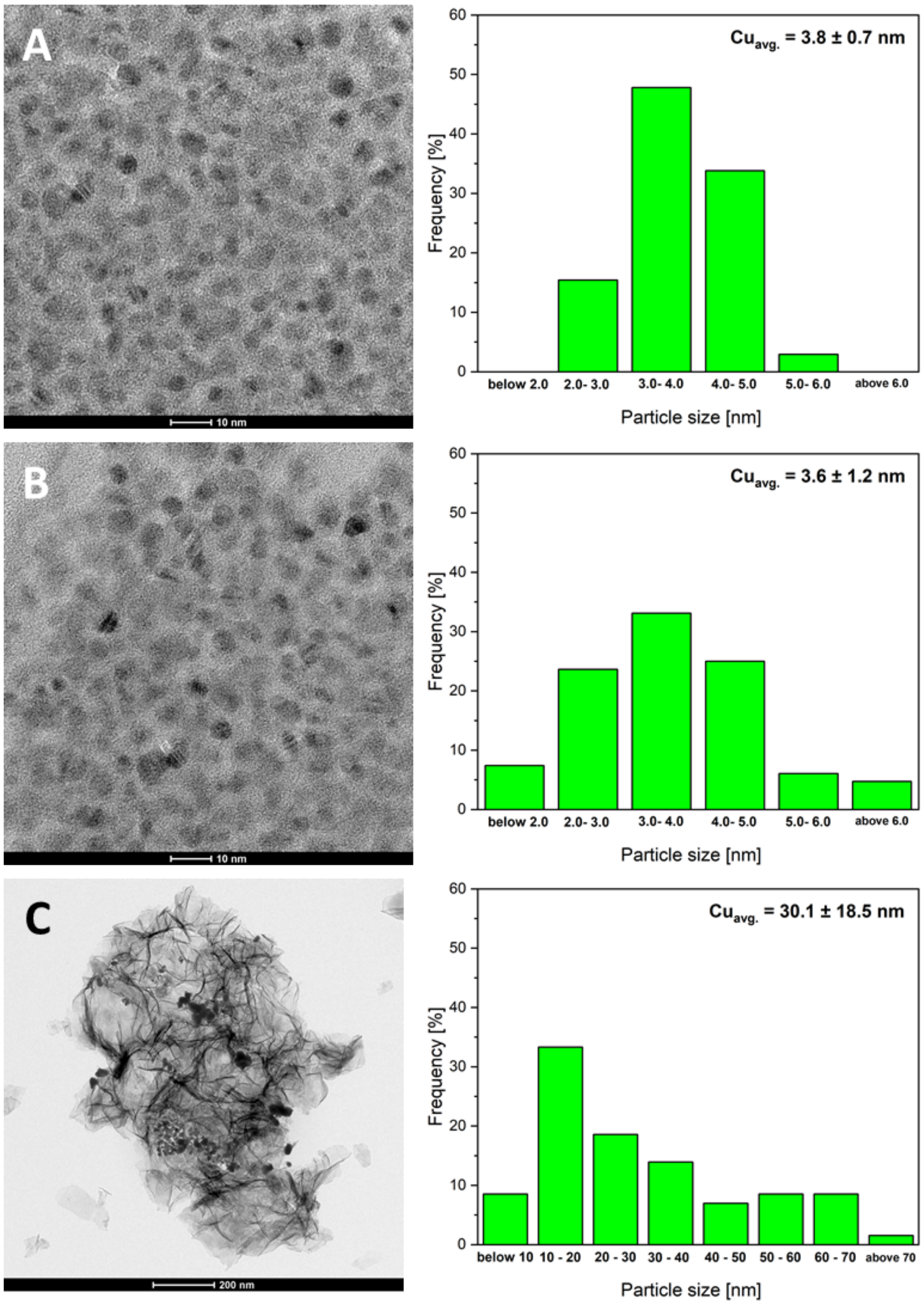

Figure S14. TEM images (left) and corresponding histograms of Cu particles size (right) for the H-S@CZMA_20mM (A) H-S@CZMA_R5 (B) and H-S@CZMA_80mM (C) catalysts.

Supporting tables

Table S1. Element analysis for S@MA-LDH core-shell and H-S@MA-LDH spheres; the numbers in brackets are the ones calculated from formula.

Sample	Formula	C[1] [wt.%]	H[1] [wt.%]	N[1] [wt.%]	Mg/Al molar ratio[2]	Si/Al molar ratio[2]	LDH[3] [wt.%]	SiO_2[3] [wt.%]
S@MA-LDH	$[SiO_2]_{0.81}$@$[Mg_{2.18}Al_{1.00}(OH)_{6.36}(CO_3)_{0.13}(OH)_{0.74}(H_2O)_{3.64}]_{0.19}$	1.58 (1.53)	3.33 (2.74)	0.00	2.18 (2.18)	3.77 (4.26)	52.00 (52.09)	48.00 (47.97)
H-S@MA-LDH	$[SiO_2]_{0.44}$@$[Mg_{2.16}Al_{1.00}(OH)_{6.32}(CO_3)_{0.25}(OH)_{0.50}(H_2O)_{0.58}]_{0.56}$	2.01 (2.01)	3.03 (3.03)	0.00	2.16 (2.16)	0.68 (0.78)	82.93 (83.19)	17.07 (17.38)

[1] Hydrogen, carbon and nitrogen content were collected from elemental analysis CHN.

[2] Mg/Al and Si/Al were collected from energy dispersive X-ray spectroscopy. The Mg/Al are close to the ones from ICP (2.27 and 2.28 for S@MA-LDH and H-S@MA-LDH, respectively).

[3] LDH and silica content were obtained from TGA.

Table S2. Crystallographic properties of the sample before and after etching.

Sample	Lattice parameters[1] [nm]		LDH basal spacing [nm]	Crystallite size[2] [nm]	
	a	*c*	d(003)	$D_{(003)}$	$D_{(110)}$
S@MA-LDH	0.305	2.306	0.769	8.1	17.0
H-S@MA-LDH	0.305	2.308	0.769	7.9	17.9

[1] $a = 2d_{110}$, $c = 3d_{003}$

[2] Crystallite size of LDH in stacking or plane direction ($D_{(003)}$ and $D_{(110)}$, respectively), calculated according to Scherrer equation: $D_{(hkl)}=0.9\cdot\lambda/(\beta\cdot\cos\theta)$, $\lambda=0.154$ nm, θ is the Bragg diffraction angle (deg.), and β is the FWHM (rad.) of the diffraction peaks.

Table S3. Textural properties of the S@MA-LDH, H-S@CZMA and H-S@CZMA-LDHs after immersion in various Cu^{2+}+Zn^{2+}-containing solutions.

Sample	S_{BET} [$m^2 \cdot g^{-1}$]	V_{micro} [$cm^3 \cdot g^{-1}$]	V_{meso} [$cm^3 \cdot g^{-1}$]	V_{total} [$cm^3 \cdot g^{-1}$]
S@MA-LDH	108	0.011	0.38	0.50
H-S@MA-LDH	224	0.008	0.66	0.89
H-S@CZMA-LDH_20mM	225	0.003	0.67	0.81
H-S@CZMA-LDH_40mM	237	0.004	0.64	0.78
H-S@CZMA-LDH_60mM	156	0.007	0.49	0.55
H-S@CZMA-LDH_80mM	113	0.004	0.37	0.46

Table S4. Selected crystallographic data of the H-S@CZMA-LDHs after immersion in various Cu^{2+}+Zn^{2+}-containing solutions.

Sample	R_{wp}[1] [%]	LDH lattice parameters[2] [nm]		LHS lattice parameters[2] [nm]		
		a	*c*	*a*	*b*	*c*
H-S@CZMA-LDH_20mM	---	0.307	2.300	---	---	---
H-S@CZMA-LDH_40mM	5.33	0.306	2.300	0.560	0.609	0.694
H-S@CZMA-LDH_60mM	6.30	0.307	2.479	0.560	0.608	0.694
H-S@CZMA-LDH_80mM	4.77	0.305	2.707	0.560	0.609	0.694

[1] Weighted residual error (weighted profile R-factor)

[2] refined lattice parameters obtained using the TOPAS software

Table S5. Element analysis for selected H-S@CZMA-LDHs.

Sample	Cu [wt.%]	Zn [wt.%]	Mg [wt.%]	Al [wt.%]
H-S@CZMA-LDH_20mM	13.90	4.60	12.03	11.03
H-S@CZMA-LDH_80mM	34.94	2.13	2.55	7.93
H-S@CZMA-LDH_R5	12.59	9.08	10.68	9.50

Cu, Zn, Mg and Al was obtained from ICP-OES.

Table S6. Comparison of reported performance of various Cu-containing catalysts with respect to the reaction conditions.

Catalyst	Temp [°C]	Pressure [bar]	CO_2 conversion [%]	MeOH selectivity [%]	STY_{MeOH} [$g_{MeOH}\cdot g_{Cu}^{-1}\cdot h^{-1}$]	Ref.
H-S@CZMA_20mM (13.9 wt.% Cu)	270	30	8.8	36.1	1.68	This work
H-S@CZMA_80mM (34.9 wt.% Cu)	270	30	1.6	69.5	0.20	This work
H-S@CZMA_R5 (12.6 wt.% Cu)	270	30	10.2	33.6	1.41	This work
Commercial $Cu/ZnO/Al_2O_3$ (35.0 wt.% Cu)	270	30	19.0	30.0	1.00	This work
LDOH-2.5Cu6Zn (4.2 wt.% Cu)	260	30	-	100.0	0.34	2
LDOH-10Cu6Zn (12.3 wt.% Cu)	260	30	-	82.2	0.90	2
$Cu-ZnO-ZrO_2$ (45.7 wt.% Cu)	250	30	7.5	59.5	0.11	3
$Cu-ZnO-ZrO_2/MgAl$ (12.2 wt.% Cu)	250	30	4.9	78.3	0.30	3
CuO/ZnO (48.2 at.% Cu)	240	30	16.1	36.5	0.10	4
$CuO–ZnO/Al_2O_3$ (3.8 wt.% Cu)	250	50	8.0	54.1	3.95	5
$CuO–ZnO/SiO_2$ (2.9 wt.% Cu)	250	50	4.0	39.9	2.41	5
CZG5Ga (31.9 wt.% Cu)	270	45	15.8	38.0	1.16	6
LDH30Ga (33.5 wt.% Cu)	270	45	19.9	48.0	1.76	6

Cu/ZnO/AlOOH (42.6 at.% Cu)	250	30	21.1	38.0	0.42	7
Cu/ZnO/Al_2O_3 (61.7 at.% Cu)	250	50	24.8	54.4	0.51	7
ZnSiO/0.2ZnCu (8.7 wt.% Cu)	220	30	1.0	100.0	0.52	8
ZnSiO/0.2ZnCu (8.7 wt.% Cu)	280	30	2.2	52.0	0.70	8
ES757@$Cu_{1.3}$ZnAl (19.8 wt.% Cu)	270	45	23	48	4.17	9
MCM-48@Cu_2ZnAl (25 wt.% Cu)	270	45	21	57	3.71	9
MSS-Si-3.2Cu-6.0Zn (3.2 wt. % Cu)	240	30	-	-	0.82	10
MSS-Si-2.2Cu-6.7Zn (2.2 wt. % Cu)	240	30	-	-	1.22	10
Cu3/LTH (Mg-Zn-Al) (18.1 wt. % Cu)	250	30	23.0	73.6	0.80	11

References:

1. T. Kondratowicz, S. Slang, L. Dubnová, O. Kikhtyanin, P. Bělina and L. Čapek, *Applied Clay Science*, 2022, **216**, 106365.
2. A. M. H. Lim, J. W. Yeo and H. C. Zeng, *ACS Applied Energy Materials*, 2023, **6**, 782-794.
3. X. Fang, Y. Men, F. Wu, Q. Zhao, R. Singh, P. Xiao, T. Du and P. A. Webley, *Journal of CO2 Utilization*, 2019, **29**, 57-64.
4. J. Xiao, D. Mao, X. Guo and J. Yu, *Applied Surface Science*, 2015, **338**, 146-153.
5. O. Tursunov, L. Kustov and Z. Tilyabaev, *Journal of the Taiwan Institute of Chemical Engineers*, 2017, **78**, 416-422.
6. M. M. J. Li, C. Chen, T. Ayvalı, H. Suo, J. Zheng, I. F. Teixeira, L. Ye, H. Zou, D. O'Hare and S. C. E. Tsang, *ACS Catalysis*, 2018, **8**, 4390-4401.
7. E. Choi, K. Song, S. An, K. Lee, M. Youn, K. Park, S. Jeong and H. Kim, *Korean Journal of Chemical Engineering*, 2018, **35**, 73-81.
8. B. Sun, A. M. Lim and H. C. Zeng, *ACS Sustainable Chemistry & Engineering*, 2023, **11**, 8326-8336.
9. M. Lyu, J. Zheng, C. Coulthard, J. Ren, Y. Zhao, S. C. E. Tsang, C. Chen and D. O'Hare, *Chemical Science*, 2023, **14**, 9814-9819.
10. Y. Shao and H. C. Zeng, *Journal of Materials Chemistry A*, 2023, **11**, 2698-2710.
11. J. Li, T. Du, Y. Li, H. Jia, Y. Wang, Y. Song and X. Fang, *Journal of Catalysis*, 2022, **409**, 24-32.
